# Supplementary material for: Association between a soy-based infant diet and the onset of puberty: A systematic review and meta-analysis
Source: PLoS One. 2021 May 18;16(5):e0251241. doi: 10.1371/journal.pone.0251241 (PMC8130953; doi:10.1371/journal.pone.0251241)
Supplement: S1 File — (DOCX) [file pone.0251241.s002.docx]

**SEARCH STRATEGY**

**Pubmed**

**1 - SOY**

1.1 - **Soy Foods**[Mesh] OR (Food, Soy) OR (Foods, Soy) OR (Soy Food) (Natto) OR (Soy Cheese) OR (Cheese, Soy) OR (Cheeses, Soy) OR (Soy Cheeses) OR (Texturized Soy Protein) OR (Protein, Texturized Soy) OR (Proteins, Texturized Soy) OR (Soy Protein, Texturized) OR (Soy Proteins, Texturized) OR (Texturized Soy Proteins) OR (Texturized Vegetable Protein) OR (Protein, Texturized Vegetable) OR (Vegetable Protein, Texturized) OR (Vegetable Proteins, Texturized) OR (Tofu) OR (Bean Curd, Soy) OR (Bean Curds, Soy) OR (Curd, Soy Bean) OR (Curds, Soy Bean) OR (Soy Bean Curd) OR (Soy Bean Curds) OR (Miso) OR (Soy Sauce) OR (Sauce, Soy) OR **Soy Milk**[Mesh] OR (Soy Beverage) OR (Beverage, Soy) OR (Beverages, Soy) OR (Soy Beverages) OR (Milk, Soy) OR **Soybeans**[Mesh] OR (Soybean) OR (Glycine max) OR (Soy Beans) OR (Bean, Soy) OR (Beans, Soy) OR (Soy Bean) OR **Soybeans Protein**[Mesh] OR (Soy Bean Proteins) OR (Dietary Soybean Proteins) OR (Soy Protein) OR (Soy Proteins) OR (Proteins, Soy) OR **Soybean oil**[Mesh] OR (oil, Soybean) OR (oils, Soybean) OR (Soybean Oils) OR (Soy Bean Oil) OR (oil, Soy Bean) OR (oils, Soy Bean) OR (Soy Bean Oils) OR (Soya Oil) OR (oil, Soya) OR (oils, Soya) OR (Soya Oils)

**2 – PUBERTY**

2.1 - **Puberty**[MeSH] OR (Puberties) OR **Puberty, precocious**[Mesh] OR (**P**uberty, delayed) OR (Delayed Puberty) OR (Early Puberty) OR (Precocious Puberty) **OR** (Idiopathic sexual precocity) OR (Familial precocious puberty) OR (Sexual Precocity) OR "**Sexual Maturation**"[Mesh] OR (Maturation, Sexual) OR (Maturation, Sex) OR (Sex Maturation) OR "Sexual Development"[Mesh] OR (Development, Sexual) OR (Sex Development) OR (Development, Sex) OR (Gonadal Disorder Maturation) OR (Pubertal Onset) OR "**Menstruation Disturbances**"[Mesh] OR (Precocious Menses) OR (Early Menses) OR (Early Menarche) OR (Precocious Menarche) OR (Disorders of Puberty) OR (Gonadotropin-Dependent Precocious Puberty) OR (Gonadotropin-Independent Precocious Puberty) OR (Isolated Precocious Thelarche) OR (Isolated Precocious Pubarche) OR (Isolated Precocious Menarche) OR Menarche[MeSH]

**EMBASE**

1. **SOY**

‘Soybean’/exp OR ‘Glycine max’ OR ‘raw soybean’ OR ‘soy bean’ OR ‘soya’ OR ‘soya bean’ OR ‘soyabean’ OR ‘soybeans’ OR ‘Tofu’/exp OR ‘bean curd’ OR ‘soy bean curd’ OR ‘soya bean curd’ OR ‘soybean curd’ OR ‘Soybean meal’/exp OR ‘soy bean flour’ OR ‘soy bean meal’ OR ‘soy flour’ OR ‘soy meal’ OR ‘soya bean flour’ OR ‘soya bean meal’ OR ‘soya flour’ OR ‘soya meal’ OR ‘soybean flour’ OR “soybean flower’ OR ‘Soybean oil’/exp OR ‘liposyn iii 10%’ OR ‘liposyn iii 20%’ OR ‘liposyn iii 30%’ OR ‘nutrilipid 10%’ OR ‘nutrilipid 20%’ OR ‘soja’ OR ‘soja bean oil’ OR ‘soja oil’ OR ‘soy bean oil’ OR ‘soy oil’ OR ‘soya bean oil’ OR ‘soya oil’ OR ‘soyacal’ OR ‘soyacal 10%’ OR ‘soyacal 20%’ OR ‘soybean fat’ OR “soybean sauce’ OR ‘travamulsion 10%’ OR ‘travamulsion 20%’ OR ‘Soybean protein’/exp OR ‘fujipuro’ OR ‘soy bean protein’ OR ‘soy protein’ OR ‘soy proteins’ OR ‘soya protein’ OR ‘soybean proteins’ OR ‘Soybean milk’/exp OR ‘milk, soya bean’ OR ‘soy beverage’ OR ‘soy drink’ OR ‘soy milk’ OR ‘soy protein milk’ OR ‘soya bean milk’ OR ‘soymilk’ OR ‘Soy food’/exp OR ‘soy foods’

**2 – Puberty**

‘Delayed puberty’/exp OR ‘puberta retarda’ OR ‘pubertas tarda’ OR ‘puberty delay’ OR ‘puberty retardation’ OR ‘puberty’ OR ‘delayed’ OR ‘puberty’ OR ‘delayed’ OR ‘retarded puberty’ OR ‘Puberty’/exp OR ‘advanced puberty’ OR ‘pseudopuberty’ OR ‘pubescence’ OR ‘Breast development’/exp OR ‘development’ OR ‘breast; thelarche’ OR ‘Menarche’/exp OR ‘first menstruation’ OR ‘menarchal age’ OR ‘menarche age’ OR ‘menarcheal age’ OR ‘menstruation onset’ OR ‘premenarche’ OR ‘puberal haemorrhage’ OR ‘puberal hemorrhage’ OR ‘Precocious puberty’/exp OR 'precocity, sexual’ OR ‘premature pubarche’ OR ‘premature puberty’ OR ‘premature thelarche’ OR ‘proeotia’ OR ‘proiotia’ OR ‘pubertas praecox’ OR pubertas precox OR ‘puberty praecox’ OR ‘puberty, precocious’ OR ‘sex precocity’ OR ‘sexual precocity’

**BVS (LILACS)**

#1 MH:"**Feijão de soja**" OR (Soja) OR (Feijão-Japonês) OR (Glycine max) OR (Grãos de Soja) OR MH: [B01.650.940.800.575.100.401.750](javascript:void(submit_GET_METHOD('013406','013406-1','hierarchic')))$ OR MH:"**óleo de soja**" OR MH:[D10.212.302.380.800](javascript:void(submit_GET_METHOD('019352','019352-1','hierarchic')))$ OR MH:[D10.212.507.800](javascript:void(submit_GET_METHOD('019352','019352-2','hierarchic')))$
 OR MH:[D10.627.700.880](javascript:void(submit_GET_METHOD('019352','019352-3','hierarchic')))$ OR MH:[D20.215.784.750.880](javascript:void(submit_GET_METHOD('019352','019352-4','hierarchic')))$ OR MH:[G07.203.300.375.400.750](javascript:void(submit_GET_METHOD('019352','019352-5','hierarchic')))$ OR MH:[J02.500.375.400.750](javascript:void(submit_GET_METHOD('019352','019352-6','hierarchic')))$ OR MH:"**Leite de soja**" OR (**Proteína de soja**) OR (**alimento de soja**) OR MH:[G07.203.100.712.500](javascript:void(submit_GET_METHOD('038405','038405-1','hierarchic')))$ OR MH:[G07.203.300.850.450.500.500](javascript:void(submit_GET_METHOD('038405','038405-2','hierarchic')))$ OR MH: [J02.200.712.500](javascript:void(submit_GET_METHOD('038405','038405-3','hierarchic')))$ OR MH:[J02.500.850.800.500.500](javascript:void(submit_GET_METHOD('038405','038405-4','hierarchic')))$

#2 MH:"**Puberdade precoce**" OR MH:[C19.391.693](javascript:void(submit_GET_METHOD('012064','012064-1','hierarchic')))$ OR MH:"**Puberdade tardia**" OR MH:[C19.391.690](javascript:void(submit_GET_METHOD('012063','012063-1','hierarchic')))$ OR MH:"**Puberdade**" OR MH: [G08.686.760](javascript:void(submit_GET_METHOD('029534','029534-1','hierarchic')))$ OR MH: [G08.686.841.374](javascript:void(submit_GET_METHOD('029534','029534-2','hierarchic')))$ OR MH:"**Menarca**" OR MH:[G08.686.760.410](javascript:void(submit_GET_METHOD('022242','022242-1','hierarchic')))$ OR MH: [G08.686.841.374.410](javascript:void(submit_GET_METHOD('022242','022242-2','hierarchic')))$

**CENTRAL Cochrane**

Date Run: 07/12/17 17:37:51.335

Description:

ID Search Hits

#1 MeSH descriptor: [Soy Foods] explode all trees 579

#2 MeSH descriptor: [Soy Milk] explode all trees 57

#3 MeSH descriptor: [Soybeans] explode all trees 462

#4 MeSH descriptor: [Soybean Proteins] explode all trees 426

#5 MeSH descriptor: [Soybean Oil] explode all trees 194

#6 #1 or #2 or #3 or #4 or #5 1178

#7 MeSH descriptor: [Puberty] explode all trees 352

#8 MeSH descriptor: [Puberty, Delayed] explode all trees 38

#9 MeSH descriptor: [Puberty, Precocious] explode all trees 65

#10 MeSH descriptor: [Menstruation Disturbances] explode all trees 1666

#11 MeSH descriptor: [Sexual Maturation] explode all trees 33

#12 #7 or #8 or #9 or #10 or #11 2107

#13 #6 and #12 1
