# Supplementary material for: Association between a soy-based infant diet and the onset of puberty: A systematic review and meta-analysis
Source: PLoS One. 2021 May 18;16(5):e0251241. doi: 10.1371/journal.pone.0251241 (PMC8130953; doi:10.1371/journal.pone.0251241)
Supplement: Supplementary file 3 [file pone.0251241.s001.docx]

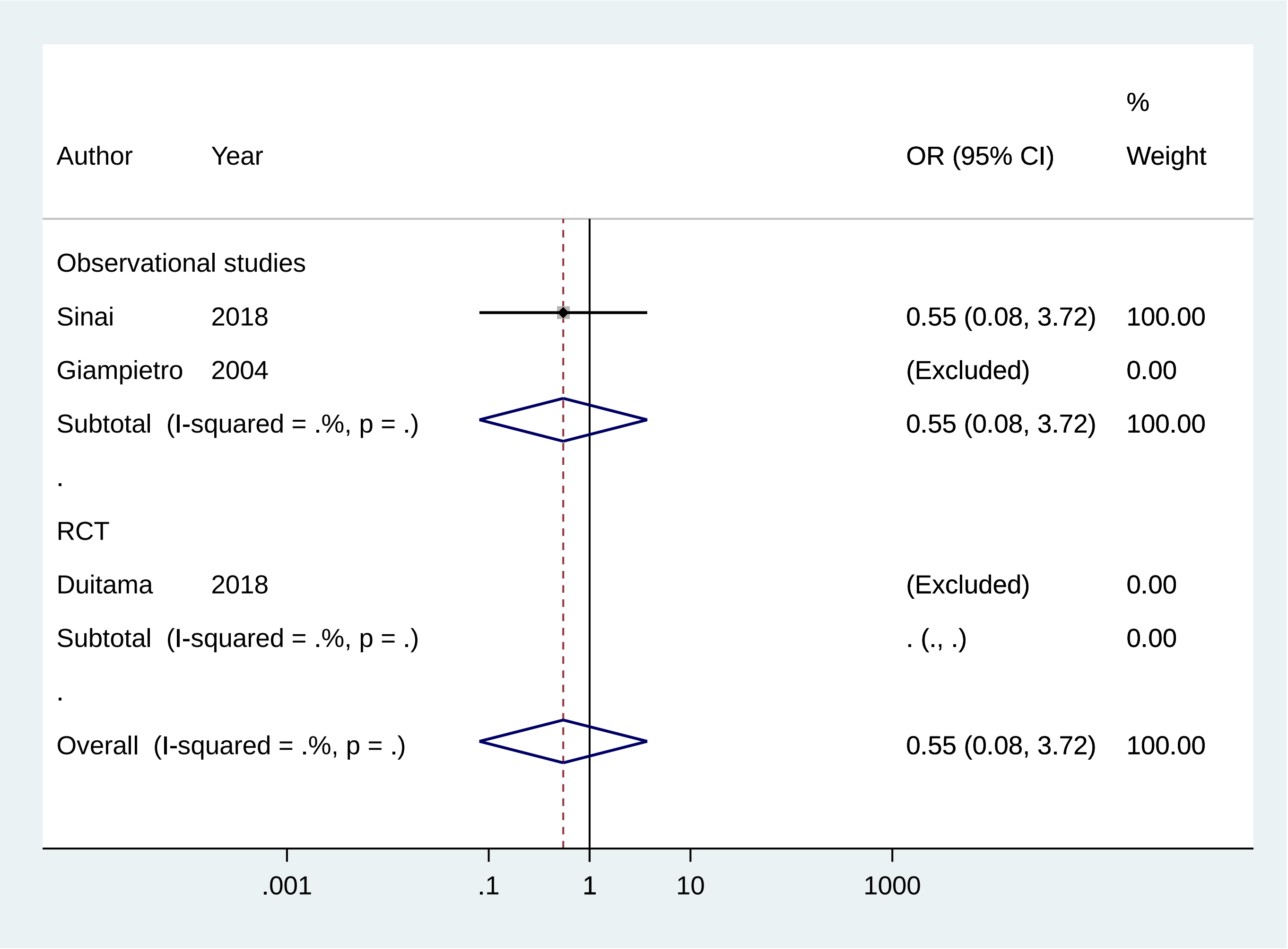
%
Author
Year
OR (95% CI)
Weight
Observational studies
Sinai 2018 0.55 (0.08, 3.72) 100.00
Giampietro 2004 (Excluded) 0.00
Subtotal (I-squared = .%, p = .) 0.55 (0.08, 3.72) 100.00
.
RCT
Duitama 2018 (Excluded) 0.00
Subtotal (I-squared = .%, p = .) . (., .) 0.00
.
Overall (I-squared = .%, p = .) 0.55 (0.08, 3.72) 100.00
.001
.1
1
10
1000
