# Supplementary material for: Association between a soy-based infant diet and the onset of puberty: A systematic review and meta-analysis
Source: PLoS One. 2021 May 18;16(5):e0251241. doi: 10.1371/journal.pone.0251241 (PMC8130953; doi:10.1371/journal.pone.0251241)
Supplement: Attachment — Submitted filename: 2 response revision.docx [file pone.0251241.s004.docx]

**Dear reviewer**

**Again, I want to like to thank you for spending time and efforts to improve our review. Your comments have improved it a lot, and we have learned many methodological aspects by your revision. Again, thank you very much.**

**1- The height outcome needs to be revisited. I notice that there are 2 studies that have reported height at measurement at beginning of puberty and one as a final adult height.**

R. Thank you for the important detail, we have emphasized the height outcome in the manuscript, line 370 to 380, and in the table 1.

**2- Table 1 should specify if the data is in Cm or SD.**

R. We have specified

**3- present the effect estimate of the reported height at measurement at beginning of puberty and one as a final adult height as separate and as combined.**

R. We have specified this in the table 3, actually only Duitama presented height at baseline and as effect estimate, the other two only presented the estimate effect

**4- convert the effect estimate to absolute data either SD or Cm rather than leaving it as SMD to improve understanding of the results.**

**R.** As Sinai et al presented data only in SD, we have excluded it from the meta-analysis. We performed meta-analysis only with Duitama e Strom, and in Strom we converted inches in centimeters, and we could perform the meta-analysis in MD (figure 4)

**5- all meta-analysis figures did not include label for the direction of the effect estimate.**

R. Sorry for this, we have included the direction of the effect estimate in the meta-analyses
 **6- across the manuscript the authors mention that they could or could not present meta-analysis figure, but the correct wording is to say that meta-analysis could or could not be performed. The figure is just an away of the results presentation, but meta-analysis is a statistical technique.**

R. Sorry for this, we have corrected this
 **7- the definition of high soy intake needs to be included in the methods section, then in the results you report what sort of intake was there int he groups and include the actual intake in table 1.**

R. Again, thank you for important observation. Unfortunately, during the protocol of this SR we could not define a high soy intake as eligibility criteria. This is because there is not an exact definition of normal soy intake in children. Additionally, our objective was to include studies who participants intake more soy than most population, as people from Asia. We have included the paragraph below in the manuscript (line 122 to 131).

Given that the mean intake of isoflavones varies from country to country and there is no definition of either a mean or high intake of this phytoestrogen in children, we did not use a cut-off point to separate participants with a high intake from those with a low intake. However, the studies were included if the authors used any tool to classify children as high or low consumers. When the mean daily intake of soy and its’ derivatives was not available, we included patients who were known to have a higher consumption of soy than the general population, such as vegetarians and Asian populations. The assessment of soy consumption was determined through dietary records, food frequency questionnaires, or the participant’s use of soy supplements.

**8- the authors need to describe how the reported findings in the risk of bias section from studies had influenced the risk of bias scores for each one. for example, did studies using self-reported pubertal data were given high or low risk of bias score? this paragraph need to be restructured as specific ROB domains, then overall.**

**R.** Actually we could apply Rob 1 only in Duitama, because the other studies were observational. However, the table 4 (Explanations) we have emphasized that this point was important to rate down the quality of evidence in the risk of bias.
 **9-the opening paragraph of the discussion need to be focused on the study findings and not to reiterate what was mentioned already in the introduction.**

R. We have removed what was mentioned already in the introduction.

**10- the GRADE table, please change to the table view " GRADE profile (V2)) to give details on judgments leading to low quality evidence since it’s not described in the text.**
R. Thank you very much for this suggestion, we have change for Grade profile V2 and we have given details on judgments leading to low quality evidence

**11- the outcome data in the risk of the outcome with no soybean, how did you come up with the numbers? are these using the risk in the general population or in the reported comparison group from included studies?
please include foot note to explain this.
for example the mean timing of menarche should not be 0, rather it should be 12,7 years**

R. We have reviewed the table of GRADE with the data from the meta-analyses in order to become it clear than the first one.
 **12- in table 1, please highlight the country of the study in a different column rather than combining it with the population, also age & BMI need to be separated into columns. All ages need to be standardized either years or months. add a column for total number of children followed in each study.**

R. We have highlighted in a different column all these data
 **13- the results are a bit confusing with regard to PP, in table 2 it says 40 out of 1000 are expected to have PP in the unexposed group vs 8 out of 1000 are expected to have PP in the exposed group. although not statistically significant effect, but it is suggesting more PP with lack of exposure, that raises suspicion about the results. Siani 2018, was the only study to report PP while the others had 0 events, the children in his study had cow’s milk allergy. It is important to understand more information about those children such as: what was the other milk types they took, for how long. was the exposure to soy milk limited to the 1st year of life?
the discussion section for such observation is important and need to highlight all these details. The current discussion is not focused on this element. please consider if leaving this study out of the analysis would be the most appropriate action because those children are considered different than typically other healthy children in terms of type of nutritional intake because of the allergy.**

R. We have updated these data in the table of GRADE. I have included all details of Sinai 2018 in the lines 251 to 254. We have included this point in the discussion section (line 447 to 452). In the meta-analysis of height Sinai was excluded due to present data only in SD. We have not excluded it from the review but emphasized this as a limitation (line 447 to 452) **14- in the limitation section please include that future studies need to report physician assessed puberty stage, and the progression of puberty in those children, in addition to: expected mid parental height, parental puberty.**

R. Okay, we have included **15- need to consider English editing**

R. Okay, we used Editage for English editing
