# Supplementary material for: Association between a soy-based infant diet and the onset of puberty: A systematic review and meta-analysis
Source: PLoS One. 2021 May 18;16(5):e0251241. doi: 10.1371/journal.pone.0251241 (PMC8130953; doi:10.1371/journal.pone.0251241)
Supplement: Attachment — Submitted filename: 3 response revision to reviewer 1.docx [file pone.0251241.s005.docx]

April 20, 2021

Dear Reviewer

We have revised the manuscript according to your comment about table 1. We have reviewed table 1 and table 2, and we used the same metrics (years) in all anthropometric data, as well as follow up time in the table 4.

Again, thank you so much for spending time reviewing and consequently improving our manuscript.

Your Sincerely,

Vania dos Santos Nunes Nogueira

Department of Internal Medicine – Botucatu Medical School – Sao Paulo State University/UNESP, Sao Paulo, Brazil. Tel: (55 14) 3880 11 71E-mail: vania.nunes-nogueira@unesp.br
